# High-Degree Middle Cerebral Artery Stenosis: Can Advanced 3D DSA-MRI Fusion Imaging Better Illustrate Plaques and Perforators?

**DOI:** 10.1007/s00062-020-00927-w

**Published:** 2020-07-02

**Authors:** Min Guan, Jia’xing Lin, Sheng’ming Huang, Xin’yi Leng, Chang’zheng Shi, Hong’yu Qiao, Xiang’yu Wang, Li’an Huang

**Affiliations:** 1 Department of Neurology, The First Affiliated Hospital, Jinan University, NO.613 the West of Huangpu street, 510630 Guangzhou, China; 2 Department of Medicine & Therapeutics, The Chinese University of Hong Kong Shatin, Hong Kong, SAR China; 3 Medical Imaging Center, The First Affiliated Hospital, Jinan University, Guangzhou, China; 4 Department of Neurosurgery, The First Affiliated Hospital, Jinan University, Guangzhou, China

**Keywords:** Middle cerebral artery stenosis, Magnetic resonance imaging, Digital subtracted angiography, Fusion imaging, Lenticulostriate arteries

## Abstract

**Purpose:**

Endovascular treatment in severe middle cerebral artery (MCA) stenosis is controversial owing to high rates of periprocedural complications, especially occlusion of the lenticulostriate arteries (LSA). The characteristics of LSAs and the spatial relationships between MCA plaques and LSAs using the fusion of three-dimensional (3D) digital subtraction angiography (DSA) and magnetic resonance imaging (3D DSA-MRI fusion) were investigated.

**Methods:**

We retrospectively analyzed data from 32 ischemic stroke or transient ischemic attack patients with severe MCA stenosis, who underwent MRI and DSA within 2 weeks after symptom onset. The patients were divided into culprit and non-culprit MCA stenosis groups. The 3D DSA-MRI fusion was performed on dedicated workstations, which allowed automated overlays of the target vessels. The characteristics of LSAs, plaque distribution and lesion patterns, and their relationships were evaluated.

**Results:**

The 3D DSA-MRI fusion image was able to illustrate the spatial relationships between MCA plaques and LSA orifices. Of 42 LSA stems in 32 patients, 10 had MCA plaque over the LSA orifice and were all found in the culprit MCA stenosis group. Over half (51.9%) of the LSA stems in patients with culprit MCA stenosis originated from the stenotic MCA segment. The MCA plaque-LSA orifice spatial relationships were classified into four types, which were significantly different between the two groups (*p* = 0.016).

**Conclusion:**

The 3D DSA-MRI fusion technique enables visualization of the LSA orifice and MCA plaque and their spatial relationships. This classification of the type of spatial relationships can provide insights into the pathogenesis of MCA stroke and useful guides for treatment strategies.

## Introduction

Intracranial atherosclerotic stenosis (ICAS) is a major cause of ischemic stroke worldwide and the middle cerebral artery (MCA) is the most common site of ICAS among Asian populations [1, 2]. Patients with high-grade (≥70%) MCA stenosis face a high risk of stroke occurrence and recurrence, despite best medical treatment [3]. The stenting and aggressive medical management for preventing recurrent stroke in intracranial arterial stenosis (SAMMPRIS) and the Vitesse intracranial stent study for ischemic stroke therapy (VISSIT) trials showed an increased risk of stroke with medical treatment plus percutaneous transluminal angioplasty and stenting (PTAS) compared with medical treatment alone, among patients with high-grade symptomatic ICAS, mostly due to the rough patient selection criteria (based solely on the severity of luminal narrowing) and high perioperative complications [4–6].

Recent registry studies indicated promising safety and efficacy of PTAS, if performed in appropriately selected patients in high-volume centers [7]. It is still challenging to determine the benefit and risk of PTAS in individual patients with symptomatic ICAS, which are probably closely related to the stroke mechanisms.

Lenticulostriate arteries (LSAs), commonly originating from the MCA M1 segment, are particularly susceptible to MCA stroke often with severe motor weakness as they supply blood to the basal ganglia and parts of the internal capsule [8]. The most common periprocedural complication of PTAS is occlusion of perforating vessel(s) near the site of stenosis, when the plaque is possibly snow-ploughed over the perforator(s) [9, 10]. In the post hoc analysis of the SAMMPRIS trial, the rate of 30-day ischemic stroke after PTAS was decreased from 14.7% to 9.4%, when excluding patients with perforator infarction [11]. Therefore, understanding the spatial relationships between LSAs and MCA plaque is important in understanding the stroke mechanisms, guiding clinical decisions regarding PTAS versus medical treatment, and determining risks of periprocedural complications of PTAS; however, so far evidence regarding their relationships is mostly from post-mortem studies rather than in vivo studies, which was investigated in the current study with coregistration of magnetic resonance imaging (MRI) and three-dimensional digital subtraction angiography (3D DSA), along with their relationships with patterns of lenticulostriate infarctions, among patients with atherosclerotic stenosis of MCA.

Fusion or coregistration of 2 stand-alone imaging modalities have been used in pretreatment or posttreatment evaluations or cerebrovascular neuronavigation in various intracranial conditions, such as arteriovenous malformations (AVMs) and dissecting aneurysms [12–17]. Previous studies showed the efficacy of DSA-MR fusion to understand the anatomical relationships between small arteries and surrounding structures [15, 18]. With DSA as the gold standard for evaluation of intracranial arteries, and MRI using the fast spin-echo techniques with isotropic 3D imaging (FSE-Cube) providing crucial and complementary anatomical information about MCA plaques, the fusion of the two could illustrate the morphologic characteristics of LSAs and the spatial relationships of LSA orifices with MCA plaques.

## Methods

Consecutive ischemic stroke or transient ischemic attack (TIA) patients with severe MCA stenosis admitted to the department of neurology at a teaching hospital from September 2015 to September 2018, who received MRI (with diffusion-weighted imaging, DWI, and FSE-Cube) and 3D DSA within 2 weeks after symptom onset, were retrospectively screened and analyzed. The study was approved by the local institutional review board for retrospective data collection and review. Subjects fulfilling the following criteria were analyzed in the current study: 1) aged 18–80 years, 2) having focal stenosis ≥70% in MCA-M1 confirmed by DSA and 3) having 1 or more vascular risk factors including hypertension, diabetes mellitus, hyperlipidemia, obesity (body mass index ≥27) and smoking. Patients were excluded if they had any of the following conditions: 1) contraindications for MRI and DSA, such as allergy to contrast agents, ferromagnetic implants or claustrophobia, 2) insufficient image quality for analysis, 3) non-atherosclerotic arterial stenosis, such as vasculitis, dissection or moyamoya disease, 4) coexistent ipsilateral extracranial or intracranial internal carotid artery stenosis >50%, 5) bilateral MCA-M1 stenosis ≥70% and 6) evidence of possible cardioembolism.

Demographic features and vascular risk factors were recorded. We divided the patients into two groups, according to whether the severe MCA stenosis was relevant to the index ischemic stroke or TIA: culprit MCA stenosis and non-culprit MCA stenosis groups. Relevance of MCA stenosis with the index ischemic event was determined by experienced neurologists; uncertainties were resolved by consulting a senior neurologist.

### Image Protocol

All MRI examinations were performed with a 3.0 T MRI scanner (GE Discovery 750, GE Healthcare, Milwaukee, WI, USA) with an 8‑channel head coil. All patients received brain MRI examination including DWI, and FSE-Cube. The FSE-Cube sequence was obtained in a coronal plane with the following parameters: repetition time/echo time (TR/TE) 800/16 ms; slice thickness, 0.6 mm; slice gap, 0.3 mm; slice number, 256; image matrix 480 × 320; field of view (FOV) 23.0 cm × 18.4 cm; voxel size = 0.48 mm × 0.57 mm × 0.6 mm and scan duration, 6 min 32 s.

All patients underwent 3D DSA examination (Siemens Artist Zeego System, Siemens AG, Forchheim, Germany) while under local anesthesia. The tip of the catheter was positioned in the common carotid artery. Standard two-dimensional (2D) DSA was initially performed; 3D rotational images were then obtained from a series of subtracted images acquired with a rotation of a vascular C‑arm [19] and contrast injection (3 ml/s × 6 s, iopamidol 300 mg/ml) with the following parameters: angle 200º, angulation step 1.5º/f, and FOV 42 cm × 42 cm. The rotational angiographic data were reconstructed using dual volume model, then the mask volume and subtraction volume were obtained.

### Fusion of 3D DSA and MR Images

**Fig. 1:**
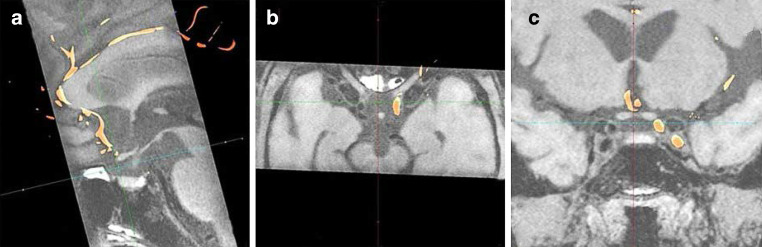
Fusion results of three-dimensional digital subtraction angiography (3D DSA) and magnetic resonance imaging (MRI). The 3D DSA and MRI are fused using a machine-based technique on the three images automatically displaying in real-time. The 3D DSA (*orange*) and MRI (*background*) fusion results of the major arteries are seen in the same window in **a** sagittal, **b** coronal and **c** axial orientations

The 3D DSA and MR images of each case were uploaded in the Siemens syngo X Workplace (Siemens AG, Forchheim, Germany). Mask volume of 3D DSA image and FSE-Cube data were fused using the post-processing automatic fusion technique. The fusion algorithm was based on the registration matched to the bony references (i.e., the skull). The method provided by the fusion software in this study was automatic registration. We assessed the quality of co-registration of 3D DSA and MRI images in the same window using axial, sagittal, and coronal views by clicking on the option called fully automatic to examine whether major arteries imaged by 3D DSA on the subtraction volume were superimposed on corresponding arteries in MR images [18]. When there was an incomplete overlap between MRI and 3D DSA of the MCA, the anterior cerebral artery and distal internal carotid artery in any of the three orientations, manual microadjustment was conducted for visual matching. Image fusion of each case was performed by two neuroradiologists (with 10 years experience in interpreting MRI and DSA images) blinded to clinical data. Disagreement was resolved by discussion. The MRI and 3D DSA images were presented with different colors in the same windows as shown in Fig. 1.

### Image Interpretation

In each case we recorded the location of infarcts and assessed features of LSAs, MCA plaque and infarct patterns, and their relationships, in fused 3D DSA and MRI images in Siemens syngo X Workplace. The origin, course and region of blood supply of LSAs were recorded. The number of LSAs originating from MCA M1 was recorded, which was approximately equal to the sum of the number of LSA stem and daughter LSAs. The LSA stem was defined as the LSA segment directly originating from the MCA; daughter LSAs were defined as subsequent branches originating from a parent LSA stem [8]. The LSAs were defined as three segments: the orifice segment (derived from the MCA-M1 trunk), cerebrospinal fluid segment (through cerebrospinal fluid and entering into the anterior perforated substance [APS]), and intracerebral segment (from APS to basal ganglia).

**Fig. 2:**
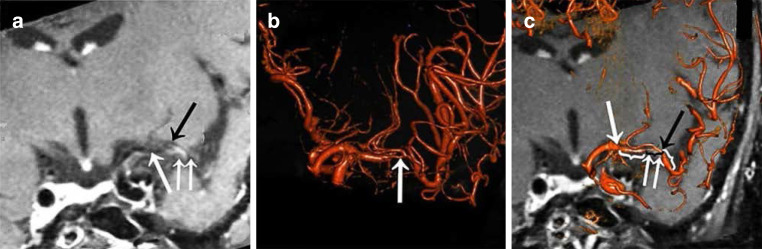
Illustration of the three-dimensional digital subtraction angiography (3D DSA) and magnetic resonance imaging (MRI) fusion imaging. **a** Coronal view of MRI, demonstrating lumen of middle cerebral artery (MCA) with low signal (*white arrow*), lenticulostriate arteries (LSAs) with low signal (*black arrow*) and plaques in MCA vessel wall with high signal (*double arrow*), which, however, traces the position of the LSA orifice difficultly. **b** The 3D DSA shows MCA stenosis and orifice of the LSAs (*white arrow*), which, however, does not reveal the spatial relationships between LSA orifice, MCA plaque, and the adjacent brain tissue. **c** The 3D DSA-MRI fusion image clearly and simultaneously displays the MCA lumen (*white arrow*) and plaque distribution (*double arrow*), the orifice of LSAs (*black arrow*), and the adjacent brain tissue. The segment labeled with the black brace is defined as the stenotic MCA segment, and the other two segments with white braces are proximal and distal non-stenotic MCA segments

We also evaluated whether plaques from MCA or in situ LSAs were present on the orifice of LSAs and whether there was LSA territory infarction. The MCA-M1 trunk was divided into 3 segments, the proximal non-stenotic segment (if the plaque was at the MCA origin the segment was excluded), the stenotic segment, and the distal non-stenotic segment (Fig. 2c). Furthermore, the relative spatial position between the MCA-M1 trunk, the orifice of LSAs and MCA plaques was classified into four types: LSAs derived from the stenotic MCA segment with the MCA plaque(s) growing over (type 1) or outside (type 2) the orifice of LSAs; LSAs derived from non-stenotic MCA segment with atherosclerotic plaque inside (type 3) or not in (type 4) the orifice of LSAs.

### Statistical Analysis

Continuous values were expressed as medians (interquartile range [IQR]), and nominal variables as numbers (percentage). Mann–Whitney *U*-tests were conducted to compare continuous variables between 2 groups; Fisher’s exact tests or χ^2^-tests were performed to compare categorical variables between 2 groups. Statistical analyses were conducted using SPSS for Windows (version 21.0, IBM, Armonk, NY, USA). A 2-sided *p* < 0.05 indicated a statistically significant difference.

## Results

### Patient Characteristics

A total of 32 patients were analyzed in the current study, 22 with culprit MCA stenosis. There was no statistically significant difference in patient demographics and main clinical characteristics between the two groups (Table 1).

**Table 1:** Baseline characteristics of patients in the two groups

Characteristics	Relevance of MCA stenosis to the index ischemic event		
	Culprit MCA stenosis (*n* = 22)	Non-culprit MCA stenosis (*n* = 10)	*p* Value
Age, years	53 (48–60)	52 (49–63)	0.984
Men	16 (72.7%)	5 (50.0%)	0.252
Hypertension	15 (68.2%)	8 (80.0%)	0.681
Diabetes mellitus	7 (31.8%)	5 (50.0%)	0.438
Hyperlipidemia	4 (18.2%)	2 (20.0%)	1.000
Obesity	4 (18.2%)	1 (10.0%)	1.000
Smoking	13 (59.1%)	5 (50.0%)	0.712
Interval between 3D DSA and MRI, days	3 (1–6)	2.5 (1–4)	0.388

*MRI* magnetic resonance imaging, *3D DSA* 3‑dimensional digital subtraction angiography, *MCA* middle cerebral artery

Values are medians (interquartile range) or numbers (percentage)

### 3D DSA-MRI Fusion Image

**Fig. 3:**
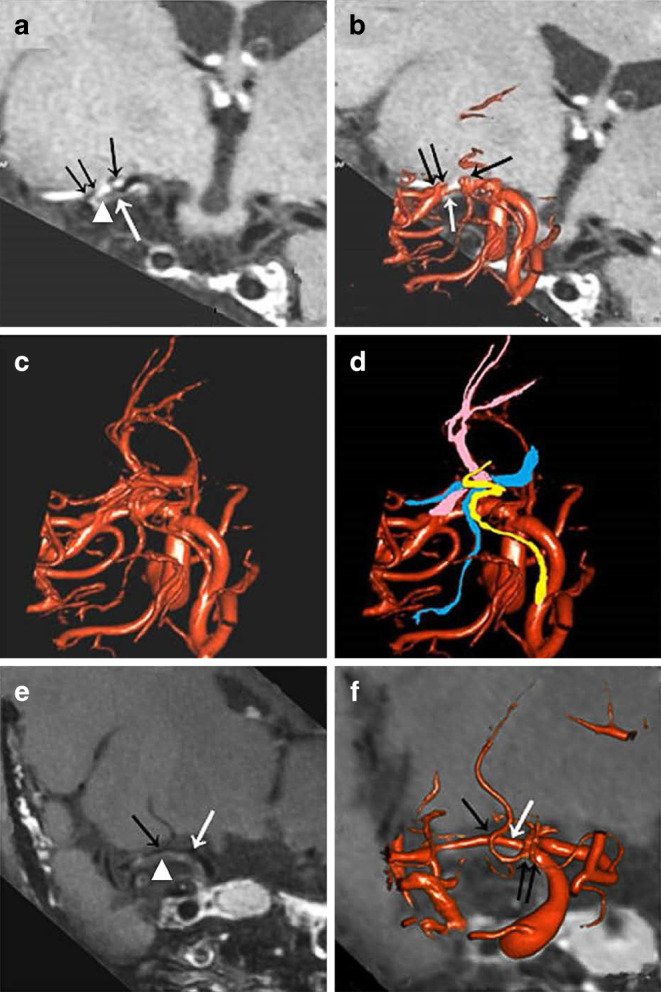
Two cases of misjudgment of the position of a lenticulostriate artery (LSA) orifice with magnetic resonance imaging (MRI). Case 1. **a,b** The MRI shows the apparent “orifice” of LSA (*black arrow*) that derived from the stenotic middle cerebral artery (MCA) segment, lumen of MCA (*white arrow*) and MCA plaques (*arrowhead*), but misjudges the true orifice of LSA (*double black arrow*). **c,d** The position of the apparent “orifice” of LSA actually consists of recurrent artery of Heubner (*yellow*) and parallel veins (*blue*) in three-dimensional digital subtraction angiography (3D DSA). The true LSA is located at the distal non-stenotic MCA segment (*pink*). Case 2. **e** The MRI shows lumen of MCA (*white arrow*), the apparent “orifice” of LSA (*black arrow*) and MCA plaques (*arrowhead*). **f** The 3D DSA and MRI fusion image confirms that the position of apparent “orifice” of LSA in MRI (*black arrow*) is the intersection of LSA and MCA (*white arrow*). The true orifice of LSA is located at a more proximal section of the MCA (*double black arrow*)

The Fig. 2 illustrates the 3D DSA-MRI fusion images in visualizing the spatial relationships between MCA plaque, LSA orifice, and adjacent brain tissue. The use of MRI alone might misinterpret the position of a LSA orifice and its spatial relationship with a MCA-M1 plaque; on the other hand, although 3D DSA could accurately reveal the position of a LSA orifice, it could not picture its spatial relationship with the MCA-M1 plaque; however, MRI and 3D DSA images were complementary in viewing these features and 2 cases of MCA stenosis are shown in Fig. 3.

### Morphological Features of LSAs

**Fig. 4:**
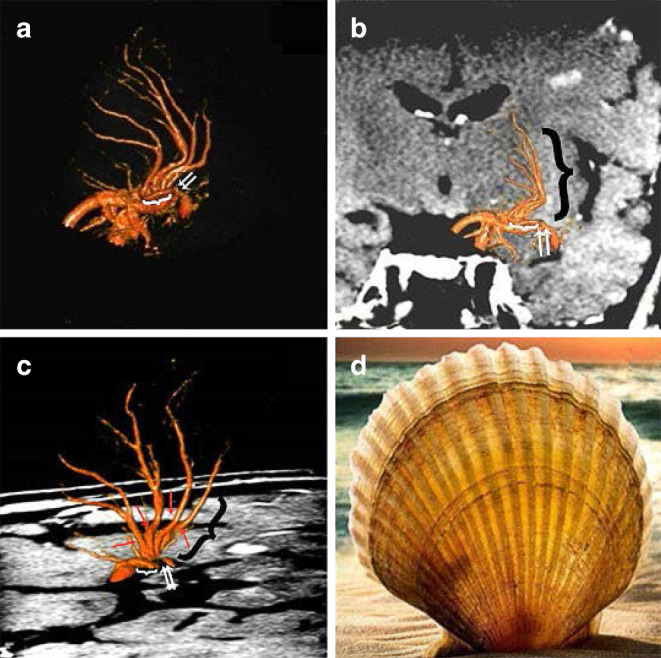
The number and the path of lenticulostriate arteries (LSAs). **a–c** The LSAs are divided into the orifice segment (*white arrows*), cerebrospinal fluid segment (*white brace*) and intracerebral segment (*black brace*). The orifice and cerebrospinal fluid segments of LSAs send out 4 daughter LSAs (*red arrows*) from a common LSA stem and the intracerebral segment bends inward in a curved shape, reaching the caudate nucleus at the end of the lenticular nucleus. **d** The path of LSAs is shaped like a shell

We recorded the number of LSAs and the path of LSA stem(s) and daughter LSAs in each case. After originating from MCA, most LSAs turned medially in close proximity to or in contact with MCA before penetrating the APS and deeper brain tissue in a zigzag course, supplying blood to different regions in the corpus striatum, globus pallidus, and internal capsule. The orifice and cerebrospinal fluid segments of LSAs sent out one or more common stems in the cerebrospinal fluid towards APS, and the intracerebral segment sent out a number of branches that reached the basal ganglia in the distal end. A representative case is shown in Fig. 4.

A comparison of the morphologic features of MCA and LSAs between culprit and non-culprit MCA stenosis groups is shown in Table 2. The degrees of MCA stenosis were similar between the two groups. A total of 131 LSAs were observed, including 42 LSA stems (27 in culprit MCA stenosis group and 15 in non-culprit MCA stenosis group) and 89 daughter LSAs. No statistical difference was seen in the total number of LSAs between the two groups (median, 4 vs. 4, *p* = 0.219), neither for the number of LSA stems (median, 1 vs. 1, *p* = 0.186) and daughter LSAs (median, 2 vs. 3, *p* = 0.344). Of the 42 LSA stems, 16 originated from the stenotic segment of the MCA, in which 10 with MCA plaques growing over the LSA origin were all detected in patients with culprit MCA stenosis; 2 LSA stems with plaques from in situ LSAs were found in culprit MCA stenosis group while 1 was in the other group. Over half (51.9%) of the LSA stem(s) in patients with culprit MCA stenosis originated from the stenotic MCA segment, while most of the LSA stem(s) in non-culprit MCA stenosis group originated from the proximal (20.0%) and distal (66.7%) non-stenotic MCA segments.

**Table 2:** Characteristics of LSAs originated from MCA between the two groups

Characteristics	Culprit MCA stenosis	Non-culprit MCA stenosis	*p* Value
Degree of MCA stenosis (%)	95 (70–99)	90 (70–99)	0.795
*Number of LSA(s) (median, interquartile range)*			
Number of LSAs	4 (3–5)	4 (3–7)	0.219
Number of LSA stem	1 (1–2)	1 (1–2)	0.186
Number of daughter LSAs	2 (2–4)	3 (2–4)	0.344
Total number of LSA stem, *n*	27	15	–
LSA stem with MCA plaques growing over LSAs orifice, *n* (%)	10 (37.0%)	0 (0.0%)	*0.020*
*Location of LSA stem, n (%)*			
Proximal non-stenotic MCA segment	2 (7.4%)	3 (20.0%)	*0.031*
Stenotic MCA segment	14 (51.9%)	2 (13.3%)	
Distal non-stenotic MCA segment	11 (40.7%)	10 (66.7%)	
Relative spatial relationships between MCA plaques and LSAs orifices, *n* (%)			
Type 1	10 (37.0%)	0 (0.0%)	*0.016*
Type 2	4 (14.8%)	2 (13.3%)	
Type 3	2 (7.4%)	1 (6.7%)	
Type 4	11 (40.8%)	12 (80.0%)	

*MCA* middle cerebral artery, *LSA* lenticulostriate arteries

### Spatial Relationships Between MCA Plaques and LSA Orifices

**Fig. 5:**
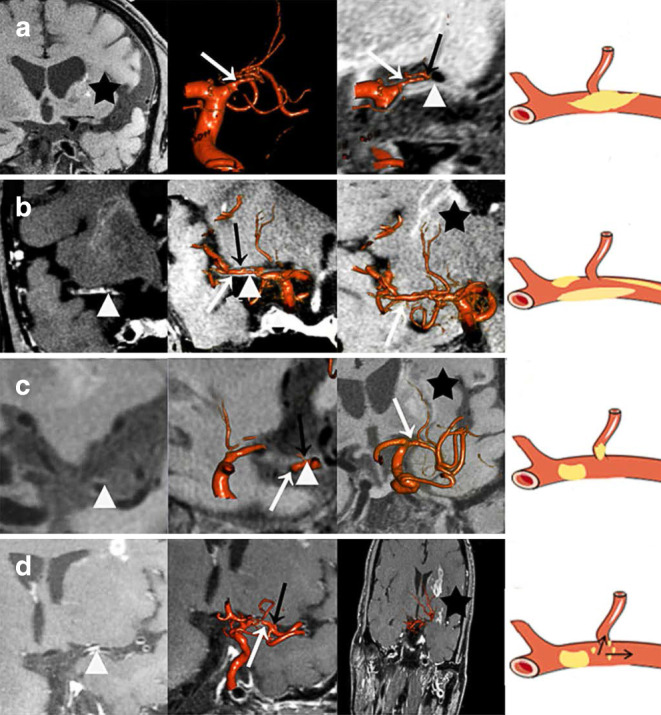
The 4 types of spatial relationships between the orifice of lenticulostriate arteries (LSAs), middle cerebral artery (MCA) plaques, and the MCA infarction topology involving the lenticulostriate region. **a** Proximal lenticulostriate infarction lesion caused by the growth of atherosclerotic plaques within the stenotic MCA segment over the orifice of LSAs (type 1); **b** distal lenticulostriate infarction lesion caused by plaques within the stenotic MCA segment and outside the orifice of LSAs (type 2); **c** proximal lenticulostriate infarction lesion mainly caused by microatheroma at the orifice of LSAs itself but not MCA stenosis (type 3) and **d** multiple infarction lesions caused by unstable plaques within MCA away from the orifice of LSAs (type 4). Of the 16 LSA stems among 13 patients with MCA infarction involving the lenticulostriate region, 7 (43.7%), 3 (18.8%), 1 (6.3%) and 5 (31.2%) had types 1–4, respectively of the relative spatial relationships between MCA plaques and LSAs orifices. The MCA lumen (*white arrow*), plaque distribution (*arrowhead*), the orifice of LSA (*black arrow*) and territory infarction (*star*); on the far right is a schematic diagram of each type

As previously mentioned the relative spatial relationships between the MCA-M1 trunk, the orifice of LSAs and MCA plaques were classified into four types, which were significantly different between those with culprit and non-culprit MCA stenosis (*p* = 0.016; Table 2). Of the 27 LSA stems among 22 patients with culprit MCA stenosis, 10 (37.0%) LSA stems derived from the stenotic MCA segment with the MCA plaque(s) growing over LSA orifices (type 1), which partly explained the infarct topology in these patients; 13 patients (59.1%) with infarction(s) involving the lenticulostriate region and 7 patients (31.8%) with infarction(s) not involving lenticulostriate region in those with ischemic stroke as the index ischemic event. The majority (80.0%) of LSA stems in patients with non-culprit MCA stenosis derived from non-stenotic MCA segment without any plaque inside the orifice of LSAs (type 4). Representative cases for the 4 types of spatial relationships between MCA-M1 trunk, the orifice of LSAs and MCA plaques, and the infarction topology are shown in Fig. 5.

## Discussion

In this study, we used the 3D DSA-MRI fusion technique to investigate the morphological features of MCA trunk, MCA plaque and LSAs in patients with high-grade MCA stenosis responsible or not responsible for an index ischemic stroke or TIA. Fused 3D DSA and MRI images accurately depicted the miniature structure and spatial relationships between these arteries/lesions. We found no difference in the degree of MCA stenosis or the numbers of LSA stem(s) and branches between those with culprit and non-culprit MCA stenosis; however, patients with culprit MCA stenosis had more LSAs originating from the stenotic MCA segment and more presence of MCA plaques growing over the orifice of the LSA(s), than those with non-culprit MCA stenosis. Moreover, we proposed a classification method for the spatial relationships between MCA-M1 trunk, the orifice of LSAs and MCA plaques, which can be used in further studies to better understand the role of such relationships in understanding the mechanisms of ischemic stroke in the presence of MCA stenosis.

The 3D DSA-MRI fusion technology shows excellent diagnostic accuracy and therapeutic guidance for neurovascular diseases as indicated in previous studies [12–15, 17]. In the current study, LSAs seemed to originate from MCA perpendicularly in MRI in some cases rather than in a zigzag course as revealed in the fusion images [20]. This would lead to misinterpretation of the true LSA orifice and its spatial relationship with MCA plaques, which is a limitation of MRI. The addition of 3D DSA could improve the accuracy in visualization of the extremely complex and numerous small vessels in the lateral fissure area, such as LSAs, recurrent artery of Heubner and parallel veins that frequently overlap and are difficult to delineate in MRI. Therefore, the 3D DSA-MRI fusion technology is promising in clinical practice and research scenarios when it is necessary to visualize these small vessels.

The path of LSAs shown in 3D DSA-MRI fusion images was consistent with previously published microanatomy findings, with LSAs usually turning medially in contact with MCA M1 after originating from MCA, and entering into basal ganglia in a curved shape afterward [16]. The total number of LSAs (median, 4) in our study was also similar to that reported in pathological studies, such as a study by Vuk Djulejic (mean 4) [21]. We also assessed features of LSA stems because the origin of the LSAs was directly related to features of MCA M1 and possibly MCA plaques. Previous pathological studies found that LSAs often arise by common stems [22] but the number of LSA stems has rarely been documented. We reported a smaller number of LSA stems (1 on average) in the current study, compared with previous imaging studies, including studies using 7.0 T time-of-flight MRA (mean, 4) and 3.0 T MRI (mean, 4) [23]. The difference may be partly attributed to the limited accuracy of MRI in illustrating LSA orifices as mentioned above, as the apparent “orifice” of LSAs might in fact be the intersections between LSA branches and MCA, which could lead to overestimation of the numbers of LSA orifices and LSA stems. It is also possible that LSAs might have been occluded by MCA plaques in the current cohort of patients with high-grade MCA stenosis. Further studies in patients with no MCA plaque or stenosis could verify such findings.

A more important finding of the current study was the differences in spatial relationships of LSA orifices with MCA trunk and plaques, between patients with the culprit and non-culprit MCA stenoses. Patients with culprit MCA stenosis had more LSA(s) originating from the stenotic MCA segment and more LSA(s) with MCA plaques growing over LSA(s) orifice, than those with non-culprit MCA stenosis. This may partly explain the mechanisms of ischemic stroke in patients with culprit MCA stenosis: first, LSAs derived from severely stenotic MCA segment maybe susceptible to hypoperfusion; second, MCA plaques partly or completely occluding the orifice of LSAs could not only lead to superficial infarction, but also deeper perforator infarctions [24] with microembolism.

The term branch atheromatous disease (BAD) had been proposed as a pathological diagnosis with atheromatous plaques at the orifice of LSAs, with the plaque from MCA or in situ LSAs [25].

However, BAD had not been visually observed in previous studies that mainly concentrated on atherosclerotic plaque characteristics and infarction features [26–28]. The 3D DSA-MRI fusion technique, however, may help to accurately subclassify the pathogenic mechanism of perforator stroke based on the relationship between plaque location and the orifice of LSAs, when BAD was equivalent to type 1 and type 3 models in the proposed classification method (Fig. 5a,c). Further studies with this classification method could provide better understanding of BAD and its association with small subcortical infarct(s) [29].

The classification method proposed in this study for the spatial relationships between MCA-M1 trunk, the orifice of LSAs and MCA plaques may also provide valuable information in selecting appropriate patients with MCA stenosis for endovascular treatment. In patients with high-grade symptomatic MCA stenosis when perforator occlusion is a probable stroke mechanism, endovascular treatment may lead to no benefit but unnecessary risks for perioperative complications [4–6]. For instance, patients with type 1 and 3 lesions might not be suitable for PTAS treatment, while type 2 and 4 lesions may benefit from PTAS with the restoration of cerebral perfusion (Fig. 5).

The current study was among the first to describe the relationships between lenticulostriate infarcts, distribution of LSAs and MCA plaques, using the 3D DSA-MRI fusion technique. This may provide insights for classification of stroke mechanisms and decision in treatment strategies in patients with symptomatic MCA stenosis; however, the study had limitations. First, there might be image distortion, such as pin cushion distortion and S distortion, in fused 3D DSA-MRI images [19] and the fusion image quality may be degraded by some artifacts from manual co-registration [30]. Second, the sample size of the study was relatively small from a single center retrospective analysis, while further longitudinal studies with larger cohorts are needed to extend applications of the 3D DSA-MRI fusion technique and explore clinical implications of our findings.

## Conclusion

The current study demonstrated the feasibility of using 3D DSA-MRI fusion technique in visualization of the miniature structural features of LSAs and MCA plaques and their spatial relationships. In patients with MCA stenosis, LSA(s) originating from the stenotic MCA segment, or LSAs with plaque growing over from MCA plaques, are more likely to be associated with infarctions in distal areas. Classification of the types of spatial relationships between MCA-M1 trunk/plaques and the orifice of LSAs, such as the classification method proposed in the current study, could help understand the stroke mechanisms and possibly guide patient selection for endovascular treatment in patients with symptomatic MCA stenosis. Further studies are warranted to verify our findings.
